# Conduction System Pacing in Pediatrics and Congenital Heart Disease: A Case Report and Literature Review

**DOI:** 10.19102/icrm.2024.15021

**Published:** 2024-02-15

**Authors:** Michael Scott, Joseph S. Needleman, Adam C. Kean

**Affiliations:** 1 Division of Pediatric Cardiology, Department of Pediatrics, Indiana University School of Medicine, Indianapolis, IN, USA; 2 Division of Pediatric Cardiology, Department of Pediatrics, Emory University, School of Medicine, Atlanta, GA, USA

**Keywords:** Cardiomyopathy, congenital heart disease, left bundle branch pacing, pacing-induced cardiomyopathy

## Abstract

Conduction system pacing involving either His bundle pacing (HBP) or left bundle branch pacing (LBBP) is a modality that has been introduced as a safe and effective alternative to right ventricular (RV) pacing to help prevent pacemaker-associated cardiomyopathy. While HBP has been employed in the pediatric and congenital populations, several small studies have shown LBBP to be safe and effective in the pediatric population. We present a patient with congenital atrioventricular block and postoperative ventricular septal defect repair cardiomyopathy with subsequent left ventricular function improvement following a transition from an RV epicardial pacemaker system to an LBBP system. This case report serves as a foundation for a review of the current state of LBBP in pediatrics and congenital heart disease.

## Introduction

His bundle pacing (HBP) and left bundle branch pacing (LBBP), collectively considered under the broader classification of conduction system pacing (CSP), have been introduced as alternatives to biventricular (BiV) cardiac resynchronization therapy (CRT). CRT has long been shown to improve BiV function in adult patients with heart failure^1^; however, this procedure is often not a practicable pacing strategy in the pediatric population.^2^ Although both HBP and LBBP have been shown to be useful in the potential prevention of pacemaker-associated cardiomyopathy, LBBP implantation may constitute a technically more successful and reliable strategy.^3,4^ Informed consent was obtained from the patient’s parents via telephone by the authors. The Indiana University/Indiana University Health Institutional Review Board deemed this case report and literature review exempt from review.

## Case presentation

**Figure 1:**
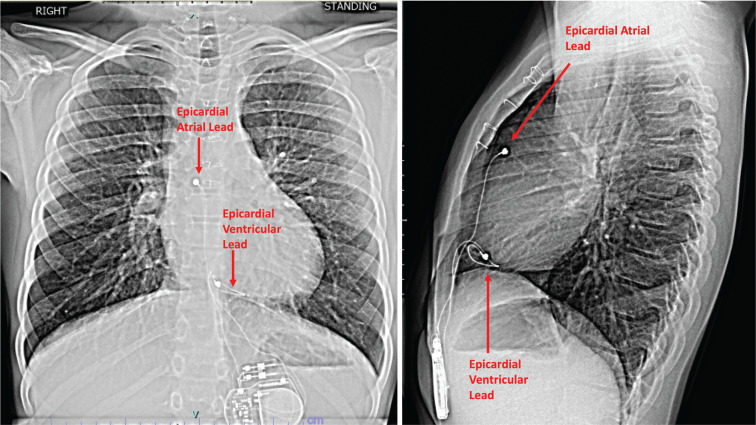
The postero-anterior and lateral chest X-ray with epicardial right ventricular pacing system 2 years prior to presentation. Note the right ventricular basal location of the ventricular lead.

A 15-year-old boy with a history including congenital atrioventricular (AV) block with secundum atrial and perimembranous ventricular septal defects (ASD, VSD) underwent single ventricular lead epicardial pacemaker placement at 2 weeks of life. His device was subsequently replaced with a dual-chamber epicardial system with DDD programming at 6 months during VSD and ASD repair. At 9 years of age, he presented with a ventricular lead fracture, underwent epicardial ventricular lead revision, and continued with DDD pacing **(Figure 1)**. His echocardiogram at that time showed mildly decreased left ventricular (LV) systolic function with an ejection fraction (EF) of 52% and a moderately dilated left ventricle. He was treated with goal-directed medical therapy, including an angiotensin-converting enzyme inhibitor/angiotensin II receptor blocker and β-blocker, as the function continued to decrease in the coming years. At 15 years of age, he had moderately decreased LV systolic function with an EF of 37% in the setting of chronic RV pacing and poor medication adherence. Despite these echocardiographic findings, he continued to be asymptomatic and played competitive soccer for his high school and club teams.

**Figure 2:**
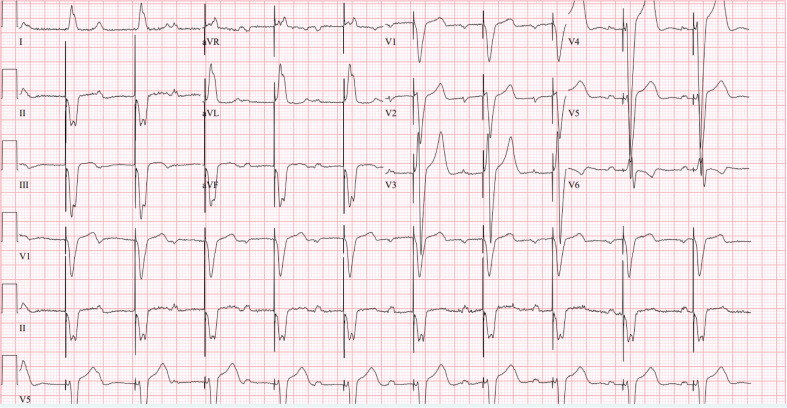
A 12-lead electocardiogram with an A-sensed, V-paced right ventricular epicardial pacing system demonstrating a left bundle branch block paced QRS morphology with prolonged QRS duration of 188 ms.

The patient subsequently presented to the emergency department with bradycardia. His device interrogation demonstrated no atrial tracking due to no sensing and an increased atrial lead impedance that was consistent with an atrial lead fracture. His device was reprogrammed from DDD 45–210 ppm to ventricular demand pacing, VVIR 60–175 ppm. At follow-up evaluation, he complained of increasingly frequent positional dizziness. He was inconsistent in his use of home medications, which included of carvedilol and losartan. His heart rate was 63 bpm, and his blood pressure was 118/58 mmHg. His physical examination revealed no evidence of congestive heart failure. Electrocardiography showed an asynchronous ventricular-paced rhythm with a QRS duration of 188 ms and a left bundle branch block (LBBB) pattern **(Figure 2)**. Echocardiography revealed mild dilation of the left ventricle with a moderately decreased EF of 37%, as well as previously noted paradoxical septal motion and no residual ASD and VSD. A device interrogation showed normal ventricular lead function with no evidence of tachyarrhythmia. Due to the setting of new-onset symptoms, progressive cardiomyopathy, and poor medication compliance, the decision was made to revise his pacing system to a transvenous system with the advantages of AV synchrony, endocardial ventricular activation, and ideally a conduction system pacing lead.

**Figure 3:**
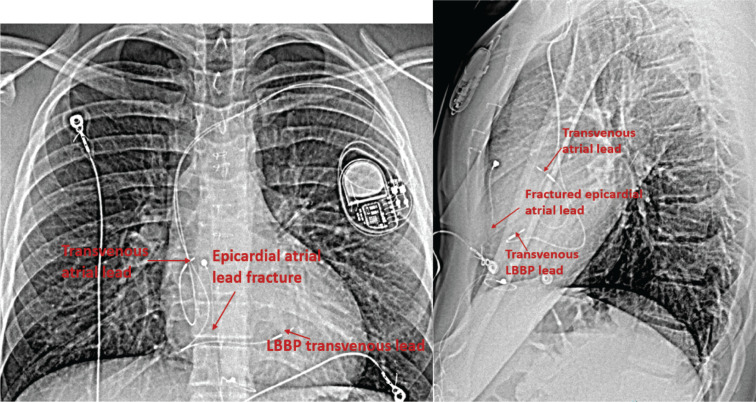
The postero-anterior and lateral chest X-ray post-transvenous left bundle branch system and atrial epicardial pacing lead fracture.

In the operating room, the patient was positioned supine and placed under general anesthesia. A transverse incision was made beneath the left clavicle, and electrocautery dissection was performed to expose the fascia above the pectoralis major. A pocket was created above the muscle with a blunt dissection. The left axillary vein was accessed using the modified Seldinger technique in two adjacent locations, and guidewires were advanced to the right atrium. A short tear-away sheath was then placed over the lateral guidewire. A SelectSite C315HIS (Medtronic Inc., Minneapolis, MN, USA) catheter was then advanced over the guidewire into the right atrium, and the guidewire was removed thereafter. A 3830-59 lead (Medtronic Inc.) lead was advanced through the catheter into the RV. There was no clear His potential when pulling back toward the tricuspid valve annulus. The catheter and lead were advanced, and the lead was then secured into the ventricular septum at the location of the left bundle. Additional lead rotations were completed under fluoroscopic guidance while maintaining catheter contact with the septum to demonstrate intraseptal lead tip location. Testing confirmed appropriate positioning of the lead with a stimulation to QRS of 41 ms and a QRS duration of 86 ms as measured by the Medtronic analyzer and the electrophysiology laboratory recording system. The SelectSite catheter was removed using the preferred spring-loaded slitting device while observing the lead tip using fluoroscopy. The short sheath was then removed without incident, and the lead was secured with a non-absorbable suture. A SelectSite C315-J (Medtronic Inc.) catheter was advanced through a separate short sheath to the right atrium, and a 3830-59 (Medtronic Inc.) lead was then advanced and secured in the right atrial appendage. Testing confirmed the position and adequate sensing and pacing thresholds. The catheter and sheath were removed, and the lead was secured. The previous abdominal generator was then removed without difficulty. The transvenous leads were subsequently connected to the generator, which was placed in the left infraclavicular pocket. A new generator (DDD 40-210) was programmed once the new leads were tested **(Figure 3)**.

**Figure 4:**
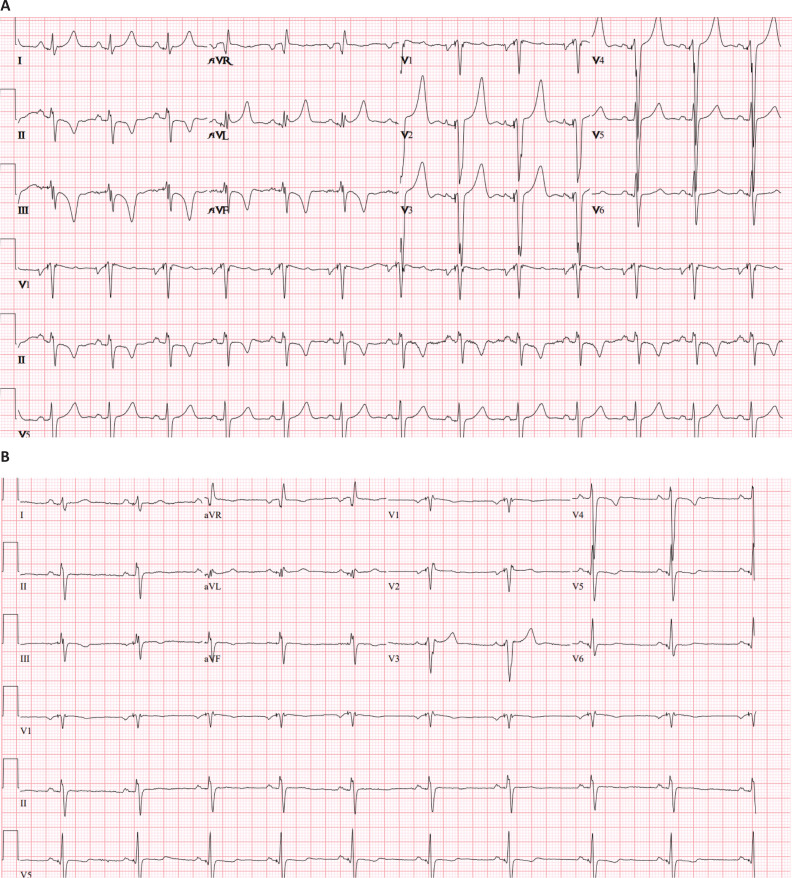
**A**: Postoperative 12-lead electocardiogram following transvenous left bundle branch system with significantly narrowed QRS duration. Note: P–R interval, 128 ms; QRS duration, 98 ms; Q–T interval, 410 ms; and axis, –61°. **B**: Follow-up electrocardiogram at 4 weeks showing right bundle branch block paced QRS morphology. Note: P–R interval, 144 ms; QRS duration, 114 ms; Q–T interval, 460 ms; and axis, –163°.

Postoperative surface electrocardiography showed an atrial-sensed ventricular-paced rhythm with a QRS duration of 98 ms **(Figure 4A)**. The patient tolerated the surgery well and was discharged the next day. At the first ambulatory follow-up evaluation, the patient reported improved energy and no orthostatic symptoms. Electrocardiography again showed an atrial-sensed, ventricular-paced rhythm with a QRS duration of 114 ms, right bundle branch block (RBBB) paced QRS morphology, and resolution of T-wave memory **(Figure 4B).** The atrial lead impedance was 532 Ω, and the ventricular lead impedance was 570 Ω. The P-wave amplitude was 5 mV, and the R-wave amplitude was >20 mV. The atrial threshold was 0.5 V at 0.4 ms, and the ventricular threshold was 0.5 V at 0.4 ms. An echocardiogram recorded just under 4 weeks postoperatively showed stable mild LV dilation with an improved EF of 48%.

## Discussion and literature review

The discussion surrounding CSP as the default strategy among pacemaker implanters continues to evolve. HBP and CSP in the pediatric and congenital heart population have shown some acceptance in the literature.^2,5^ We reviewed the current literature, focusing on the evolution of LBBP in adult and pediatric patients with and without congenital heart disease (CHD). A literature search was performed with the assistance of a medical librarian using the Medline (Ovid) database with keywords: “selective OR left OR bundle OR pacing” AND “cardiomyopathy outcomes.” These same parameters were used to complete an independent literature search in PubMed to identify additional studies missed during the first search.

### Cardiac resynchronization therapy and conduction system pacing

Chronic RV pacing is a known risk factor for pacing-associated cardiomyopathy in the pediatric population.^6–9^ CRT has been shown to ameliorate cardiomyopathy in the adult population.^1^ However, this pacing strategy is not always feasible in the pediatric population due to patients’ size and congenital malformations.^2^ Dubin et al. studied CRT in the pediatric population, and, although systolic function improved, there were more technical adverse events recorded, including dissection, perforation, or lead dislodgement associated with cardiac venous leads, when compared to adults.^10^ There was also a wide variability in technical approaches, with half of the devices being epicardial or mixed (epicardial/endocardial) approaches versus transvenous placement.^11,12^ Reasons for using an epicardial lead system versus a transvenous approach include tricuspid valve abnormalities, intracardiac shunting, and venous anomalies.^10^

The CSP has been shown to be non-inferior to BiV pacing (BVP) in adults with a CRT indication.^13^ However, a recent study by Vijayaraman et al. showed that the primary outcome rate of developing heart failure hospitalization or time to death was significantly lower in the left bundle branch area pacing (LBBAP) group compared to the BVP group.^14^ Gordon et al. reported the largest case series of CSP in patients of pediatric age in 2022.^4^ They observed improvements in pacemaker-associated cardiomyopathy following implantation of LBBP devices. This study included 24 patients (12 with CHD and 12 without) who underwent physiologic pacing with a conduction system lead. Four patients had decreased EFs of 32%–45% prior to physiologic pacing and showed intraoperative improvements of 5%–10% that persisted on subsequent serial echocardiograms. Patients were followed for a median of 610 days in this study. The results suggest an improvement in EF with the use of CSP; however, because the study was retrospective, it is indeterminate if CSP can prevent pacing-associated cardiomyopathy. Relative to HBP, selective LBBP systems appear to be simpler to implant, have increased generator longevity due to lower pacing thresholds, and have a greater amplitude of sensed R-waves, making pacing management easier.^3^ As congenitally corrected transposition of the great arteries (CCTGA) is known to be associated with the AV block, patients with this condition often require permanent pacemakers. CCTGA has been specifically well studied regarding alternatives to morphologic LV pacing.^14–19^ These studies have shown both HBP and LBBP to be safe and effective in the setting of CCTGA.

### Procedure: left bundle branch pacing

The first and only lead approved for LBBP in bradycardia patients in the United States is the SelectSecure MRI SureScan Model 3830 (Medtronic Inc.).^20^ Stylet-driven pacing lead systems from Abbott Laboratories (Chicago, IL, USA), Boston Scientific (Marlborough, MA, USA), and Biotronik (Berlin, Germany) have also been used for LBBP.^21^ There are several reported methods for placing LBBP leads. One approach involves using three-dimensional mapping catheters designed for electrophysiology studies. In this approach, an octopolar or decapolar catheter is generally used to mark the location of the His bundle by identifying the His bundle electrogram and annotating the catheter position using fluoroscopy and/or three-dimensional mapping software. The catheter may be placed via the femoral vein or right axillary vein. Once this location is identified, the lead is positioned just distal to this signal.^4^ Of note, some operators prefer to place the catheter deeper in the RV and complete at least 10 turns of the 3830 lead instead of the manufacturer-recommended 4–6 turns.^12^ Other methods include using fluoroscopy and electrocardiography only with or without the use of premature ventricular contraction morphology. The LBB area is located generally 10–20 mm from the His potential location toward the ventricular apex. The lead is positioned in the RV septum along the line between the HBP site and the RV apex in the right anterior oblique (RAO) 30° view. Pacing at high output is performed to evaluate capture prior to rotating the lead deep into the septum.^2^ Preshaping of the C315 catheter to better reach the His bundle may be advantageous for patients of smaller statures.^4^ Deflectable catheters are also available for this purpose. As noted above, stylet-driven leads have been used to help guide the lead to the left bundle area as well.

Criteria to confirm LBB capture, adapted from the work of Hua et al.,^22^ include the following:

1. Paced QRS morphology with an RBBB-like pattern
2. Recording of an LBB potential, abrupt shortening of R-wave peak times in V_6_ by >10 ms at high output pacing during lead advancement from the mid-septal region to the LV endocardium and then remaining short (usually ≤80 ms) and constant at high and low output
3. Demonstration of capture

If criterion 1 in conjunction with either criterion 2 or criterion 3 is met, then the procedure is considered successful.

### Application of left bundle branch pacing in adults

The LBBP was first clinically described by Huang et al. in 2017. In this case report, a 72-year-old patient with heart failure and dilated cardiomyopathy experienced an EF improvement from 32% to 62% at 1 year of follow-up.^23^ Subsequent research has supported long-term improvements in symptoms and EF recovery with LBBP.^24^ Tan et al. performed a meta-analysis of LBBP in the adult population in which the Preferred Reporting Items for Systematic Reviews and Meta-Analyses guidelines for systematic review were used. The study only included LBBP in patients with a CRT indication. The primary outcomes consisted of QRS duration after CRT device implantation, LV end-diastolic diameter, the New York Heart Association classification, and LVEF. Eight studies with a total of 527 patients meeting the criteria for LBBP for CRT were reviewed. The meta-analysis showed that LBBP provided physiological pacing with narrower QRS durations in patients with LBBB and heart failure with reduced EF when compared to BVP.^25^ Ravi et al. performed a single-center study evaluating LBBP performance and lead-related complications during follow-up. A total of 59 patients were reviewed in this retrospective cohort study, and the most common indication for LBBP lead implantation was intraoperative high HBP lead thresholds. Overall, there were seven lead-related complications, and three patients required lead revision, including two with macrolead dislodgements and one with an interventricular septal perforation. The latter lead was successfully explanted under transesophageal echocardiography guidance, and a new LBBP lead was implanted. Overall, 97% of patients had successful implantation of LBBP systems.^26^ None of the studies that were reviewed included a randomized controlled trial.

### Left bundle branch pacing in pediatrics and children with right ventricular pacing–associated cardiomyopathy

LBBP has also been shown to be an option in pediatric patients with congenital heart block both with and without additional structural CHD.^27–29^ A recent report highlighted a patient with hypertrophic cardiomyopathy and a subcutaneous implantable cardiac defibrillator who developed complete AV block and underwent subsequent successful implantation of an LBBP system with a separate generator.^30^ Wenlong et al. studied 12 pediatric patients who underwent implantation or replacement of an LBBP pacemaker.^31^ Eleven of the children had complete AV block, and four cases were secondary to CHD. One patient had cardiac dysfunction secondary to apical RV pacing requiring CRT. The study was completed over a 2-year period. All 12 patients underwent successful implantation of an LBBP device, and the patient with apical RV pacing cardiomyopathy showed improved EF following LBBP. A more recent retrospective study compared children with complete AV block to assess postoperative outcomes of those with RV pacing versus LBBP.^32^ A total of 48 children were studied and LBBP produced a narrow QRS duration and improved ventricular synchrony as well as a decrease in the time-to-peak longitudinal strain and the average interventricular mechanical delay time postoperatively.

LBBP and HBP have been studied together in the pediatric CHD population, but the modalities themselves were not directly compared.^12^ Jimenez et al. studied eight pediatric patients who underwent CSP with LBBP or HBP and noted a reversal of pacemaker-induced cardiomyopathy in all patients. Patients were not followed beyond initial follow-up, and further long-term studies are needed.

### Left bundle branch pacing versus His bundle pacing

With the advent of multiple types of CSP as an alternative to traditional CRT, further long-term studies directly comparing the strategies will be instructive. As previously discussed, limitations of HBP include lead instability/dislodgment and progressive increases in pacing thresholds that may be associated with loss of capture after implantation.^33^ We believe that the difficult technical aspects of HBP and postoperative complications make LBBP a better option for most pediatric patients, including those with CHD. As technology advances and comfort and familiarity in placing these systems grows, further research comparing the two modalities is warranted. Given the small volume of implanted pacing systems in children and patients with CHD, conducting large trials to answer management issues (as is common in the adult literature) is not generally practicable in the pediatric and congenital heart electrophysiology community. A multicenter registry among pediatric heart centers may offer a viable means to serve this purpose.

## Conclusion

While HBP has been employed in the pediatric and congenital populations, several small studies have shown LBBP to be safe and effective in the pediatric population. LBBP may represent a more compelling strategy with improved lead characteristics while maintaining improved LV function offered by CSP in pediatric and CHD patients. A multicenter pediatric and CHD registry may help to explore optimal pacing strategies.

## References

[1] Abraham WT, Fisher WG, Smith AL (2002). MIRACLE Study Group. Multicenter InSync Randomized Clinical Evaluation. Cardiac resynchronization in chronic heart failure. N Engl J Med.

[2] Lyon S, Dandamudi G, Kean AC (2020). Permanent His-bundle pacing in pediatrics and congenital heart disease. J Innov Card Rhythm Manag.

[3] Zhang S, Zhou X, Gold MR (2019). Left bundle branch pacing: JACC review topic of the week. J Am Coll Cardiol.

[4] Gordon A, Jimenez E, Cortez D (2022). Conduction system pacing in pediatrics and congenital heart disease, a single center series of 24 patients [published online ahead of print June 9, 2022]. Pediatr Cardiol.

[5] Chubb H, Mah D, Dubin AM, Moore J (2023). Conduction system pacing in pediatric and congenital heart disease. Front Physiol.

[6] Gebauer RA, Tomek V, Salameh A (2009). Predictors of left ventricular remodelling and failure in right ventricular pacing in the young. Eur Heart J.

[7] van Geldorp IE, Vanagt WY, Prinzen FW, Delhaas T (2011). Chronic ventricular pacing in children: toward prevention of pacing-induced heart disease. Heart Fail Rev.

[8] Kaltman JR, Ro PS, Zimmerman F (2008). Managed ventricular pacing in pediatric patients and patients with congenital heart disease. Am J Cardiol.

[9] Czosek RJ, Gao Z, Anderson JB, Knilans TK, Ollberding NJ, Spar DS (2021). Progressive QRS duration and ventricular dysfunction in pediatric patients with chronic ventricular pacing. Pediatr Cardiol.

[10] Dubin AM, Janousek J, Rhee E (2005). Resynchronization therapy in pediatric and congenital heart disease patients: an international multicenter study. J Am Coll Cardiol.

[11] Sharma PS, Dandamudi G, Naperkowski A (2015). Permanent His-bundle pacing is feasible, safe, and superior to right ventricular pacing in routine clinical practice. Heart Rhythm.

[12] Jimenez E, Zaban N, Sharma N (2020). His bundle and left bundle pacing in pediatrics and congenital heart disease: a single center experience. Pediatr Cardiol.

[13] Pujol-Lopez M, Jiménez-Arjona R, Garre P (2022). Conduction system pacing vs biventricular pacing in heart failure and wide QRS patients: LEVEL-AT trial. JACC Clin Electrophysiol.

[14] Vijayaraman P, Ponnusamy S, Cano Ó (2021). Left bundle branch area pacing for cardiac resynchronization therapy: results from the International LBBAP Collaborative Study Group. JACC Clin Electrophysiol.

[15] Kean AC, Kay WA, Patel JK, Miller JM, Dandamudi G (2017). Permanent nonselective His bundle pacing in an adult with L-transposition of the great arteries and complete AV block. Pacing Clin Electrophysiol.

[16] Moore JP, Gallotti R, Shannon KM (2020). Permanent conduction system pacing for congenitally corrected transposition of the great arteries: a pediatric and Congenital Electrophysiology Society (PACES)/International Society for Adult Congenital Heart Disease (ISACHD) Collaborative Study. Heart Rhythm.

[17] Mahata I, Macicek SL, Morin DP (2019). Direct His bundle pacing using retrograde mapping in complete heart block and L-transposition of the great arteries. HeartRhythm Case Rep.

[18] Vijayaraman P, Mascarenhas V (2019). Three-dimensional mapping-guided permanent His bundle pacing in a patient with corrected transposition of great arteries. HeartRhythm Case Rep.

[19] Takemoto M, Nakashima A, Muneuchi J (2010). Para-Hisian pacing for a pediatric patient with a congenitally corrected transposition of the great arteries (SLL). Pacing Clin Electrophysiol.

[20] Lyons K Medtronic first to receive FDA approval for pacing the heart’s natural conduction system.

[21] De Pooter J, Wauters A, Van Heuverswyn F, Le Polain de Waroux JB (2022). A guide to left bundle branch area pacing using stylet-driven pacing leads. Front Cardiovasc Med.

[22] Hua W, Gu M, Niu H, Gold MR (2022). Advances of implantation techniques for conduction system pacing. JACC Clin Electrophysiol.

[23] Huang W, Su L, Wu S (2017). A novel pacing strategy with low and stable output: pacing the left bundle branch immediately beyond the conduction block. Can J Cardiol.

[24] Huang W, Su L, Wu S (2019). Long-term outcomes of His bundle pacing in patients with heart failure with left bundle branch block. Heart.

[25] Tan JL, Lee JZ, Terrigno V (2021). Outcomes of left bundle branch area pacing for cardiac resynchronization therapy: an updated systematic review and meta-analysis. CJC Open.

[26] Ravi V, Hanifin JL, Larsen T, Huang HD, Trohman RG, Sharma PS (2020). Pros and cons of left bundle branch pacing: a single-center experience. Circ Arrhythm Electrophysiol.

[27] Dai CC, Dai WL, Guo BJ (2020). Clinical observation on six children of left bundle branch area pacing. Zhonghua Er Ke Za Zhi.

[28] Ponnusamy SS, Arora V, Namboodiri N, Kumar V, Kapoor A, Vijayaraman P (2020). Left bundle branch pacing: a comprehensive review. J Cardiovasc Electrophysiol.

[29] Ponnusamy SS, Muthu G, Bopanna D (2020). Selective left bundle branch pacing for pediatric complete heart block. Indian Pacing Electrophysiol J.

[30] Perin F, Molina-Lerma M, Jiménez-Jáimez J, Rodríguez-Vázquez Del Rey MDM, Ortega Á, Álvarez M (2021). Combined use of subcutaneous implantable defibrillator with endovenous left bundle branch pacing in a child with hypertrophic cardiomyopathy. Rev Esp Cardiol (Engl Ed).

[31] Wenlong D, Baojing G, Chencheng D, Jianzeng D (2022). Preliminary study on left bundle branch area pacing in children: clinical observation of 12 cases. J Cardiovasc Electrophysiol.

[32] Li J, Jiang H, Cui J Comparison of ventricular synchrony in children with left bundle branch area pacing and right ventricular septal pacing [published online ahead of print January 5, 2023]. Cardiol Young.

[33] Vijayaraman P, Naperkowski A, Subzposh FA (2018). Permanent His-bundle pacing: long-term lead performance and clinical outcomes. Heart Rhythm.

